# Supplementary material for: Are carbon emissions trading and green financial instruments synergistic? -Comprehensive quantitative research based on content analysis
Source: PLoS One. 2024 Mar 7;19(3):e0298601. doi: 10.1371/journal.pone.0298601 (PMC10919601; doi:10.1371/journal.pone.0298601)
Supplement: S1 Data — (ZIP) [file pone.0298601.s001.zip › Data/地级市Z_F工作报告.zip!/╦─┤¿/╫╘╣▒╩╨/└┤╫╘╣1⁄2╓┌║┼í╛┬φ┐╦╩2╛▌═°í┐_2014.docx]

各位代表：

　　我代表市政府向大会报告工作，请予审查，并请各位政协委员和其他列席人员提出意见。

　　2013年工作回顾

　　2013年是全市凝心聚力、克难奋进、跨越台阶的一年。一年来，面对复杂严峻的宏观经济形势，市政府在省委、省政府和市委的坚强领导下，在市人大、市政协的监督支持下，团结带领全市人民，紧紧围绕建设“五个自贡”、推动次级突破加快崛起，着力投资拉动产业支撑，加快“两化”互动统筹城乡发展，切实保障改善民生，全市经济社会保持平稳较快发展，主要经济指标增速高于全省、领先川南。全年地区生产总值实现1001.6亿元、增长11.3%；地方公共财政收入完成38.33亿元、增长16.2%。

　　过去一年，我们积极应对下行压力，全力保增长、促跨越，经济总量迈上千亿台阶

　　投资持续快速增长。坚持主抓投资，制定23项扩投资稳增长措施，全社会固定资产投资完成531.61亿元、增长22.2%。项目推进力度加大，建立了重大项目VIP服务室，率先在全省推行“三级联办五级协调”审批服务，每季度组织项目集中开工，247个市级重大项目完成投资298.8亿元，70个项目投产投用。项目争取成效明显，14个项目纳入省重点项目，全年到位项目补助资金12.2亿元。要素保障更加有力，协调落实项目信贷投放20亿元，争取建设用地计划1.38万亩，实施征地拆迁57.5万平方米，较好地满足了项目需要。

　　工业经济平稳运行。针对宏观经济下行影响，出台33项稳增长措施，规模以上工业增加值完成476.5亿元、增长11.5%。战略性新兴产业发展迈出新步伐，东锅公司大容量、高参数循环流化床锅炉洁净煤燃烧技术走在世界前列，旭阳药业现代中药产业园一期工程建成运行，海川公司燃机配件制造、中天胜公司1万吨特种聚酰亚胺树脂及复合材料产业化等项目加快推进，战略性新兴产业增加值占比达到28%。园区建设加快推进，“一带一核四园三区”布局更加完善，园区新增承载能力6平方公里，新开工项目43个，实现主营业务收入980亿元，成佳工业园首批项目落地。企业分类培育力度加大，发展省级产业联盟2个，25户企业列入省上市培育后备库，新培育省“小巨人”和“成长型”企业41户，新增30亿元企业2户、10亿元企业3户，新增中国驰名商标2件、国家地理标志保护产品5个。

　　服务业加快发展。加强服务业载体建设，出台13项促进消费措施，服务业增加值实现283.57亿元、增长11.5 %，成功列入全国家政服务业试点市、省级现代服务业综合改革试点市。川南公路运输物流市场和舒坪汽车货运站主体完工，川南汽贸产业园、川南五金城、西南（自贡）无水港、华商国际城、卧龙湖国际盐泉生态城等重大项目加快推进。第19届自贡灯会观灯游客和综合效益再创历史新高，乡村休闲旅游业快速发展，旅游综合收入增长18.2%。组织开展汽车展销、房交会等促销活动，汽车、商品房消费大幅增长，体育健身、文化娱乐、网络购物等消费快速发展，社会消费品零售总额完成380.24亿元、增长14.2%。

　　农业增产增效。出台18项措施，力促农业增效、农民增收，粮食连续7年丰收，农业增加值实现119.42亿元、增长3.8%。优质蔬菜、畜禽、林果、水产等特色基地快速发展，7个现代农业示范园加快建设，华西希望特驱集团纯种猪良种繁育场建成，农业标准化、规模化水平不断提升。荣县、富顺农产品加工园建设有序推进，龙都茶叶科技产业园等项目建成投产，新发展省级龙头企业3户、专合组织14户，新创国家级、省级农产品知名品牌25个。

　　过去一年，我们着力推进新型城镇化，突出强基础、提品质，发展条件不断优化

　　重大基础设施建设实现新突破。交通建设投资突破50亿元，成自泸、乐自高速公路全线通车，川南一小时通达全面实现，自隆、内威荣高速公路建设加快推进，成自泸高速富顺连接线竣工通车、大安连接线开工建设，乐自高速荣县连接线竣工通车、自流井连接线及互通立交开工建设。水利建设投资突破30亿元，小井沟水利工程坝体填筑和移民对接安置有序推进，狸狐洞水库开工大坝主体工程。110千伏荣县城北等输变电工程建成投运。市燃气储配站主体完工。

　　城镇建设步伐加快。城市总体规划调修基本完成，多规衔接、城乡统筹的全域规划体系基本构建。中心城区南部、西部、东北部新城开发有序实施，南岸科技新区、卧龙湖旅游度假区基础设施建设全面启动，东盐都大道竣工通车，时代大道东段、汇园路等道路加快建设。新一轮棚户区改造拉开帷幕，东兴寺、西山路片区综合改造实质性启动，自流井老街、吉成井片区改造有序推进。荣县、富顺县城加快建设，S305富顺绕城线A段竣工通车，荣县迈入中等城市行列，4个小城镇列入省示范小城镇。全市新增建成区面积6.3平方公里，城镇化率达到45.5%。城市管理不断加强，数字化城管实现全覆盖，夜市摊点整治成效明显，重要节点缓堵保畅初见成效。

　　生态环境持续改善。大力实施蓝天、清水、绿地、宁静、利废“五大行动”，成功创建国家园林城市。釜溪河复合绿道示范段建成开放，盐都植物园、汇东公园、龙湖公园主体完工，时代大道、东盐都大道等道路绿化基本完成，新增城市绿地220公顷，人均公园绿地达11平方米，全市森林覆盖率提高到31.4%。节能减排深入推进，关停5户污染企业，淘汰7户企业落后产能，川南首个垃圾焚烧发电项目、荣县垃圾处理厂建成投用，乡镇生活污水处理设施加快建设，城乡生活垃圾无害化处理率分别达92%和65%。小井沟、双溪水库饮用水源地生态功能区保护和建设控制规划编制完成，釜溪河水质稳步改善。

　　新村建设加快推进。围绕“四态一体、共融发展”连片推进新农村建设，实施新农村综合体项目12个，建成农民集中居住区房屋2342套、新村聚居点54个，改扩建生态庭院型新农家大院78处，集居区基础设施和公共服务配套更加完善，7.6万户农民生活条件明显改善，农民集中居住率达46.6%。21座病险水库除险加固、小农水等项目全面完成，新增和改善灌溉面积9.56万亩。实施金土地项目9个，增加耕地1.14万亩。建成高标准粮田6.5万亩。新建和改建通乡公路156公里、通村公路388公里。改造农村电网209公里。

　　过去一年，我们着力强化创新驱动，坚持抓改革、促开放，发展活力不断增强

　　科技创新成效明显。创新平台建设进一步加强，四川盐产业技术研究院、浙江大学自贡创新中心成功组建，大西洋焊接技术中心升级为国家级企业技术中心。实施国家、省重点科技项目72项，完成产业关键技术攻关项目16项、重点技术成果转化项目17项，2个项目获省科技进步一等奖，东锅公司霍锁善成为我市首位省科技杰出贡献奖获得者。专利创造取得突破，首家专利经营公司组建营运，专利申请量突破1000件、授权量达640件，均创历史新高。自贡被列为国家技术创新工程试点示范市和国家知识产权试点城市。

　　各项改革有序推进。统筹城乡综合配套改革深入实施，以确权为基础的农村生产要素开始激活，深化“双挂钩”试点获国土资源部批准，农村股份合作向多元化发展，全市规范流转土地35万亩、林权8.3万亩。企业改革继续深化，纺织供销公司等5户企业实现破产终结，17户煤矿企业关闭及兼并重组基本完成。财税改革有序推进，“营改增”试点启动实施。投融资改革成效明显，区县土地储备中心组建运营，高新投资公司10亿元城投债券、5户企业区域集优票据成功发行，自贡市商业银行实现增资扩股，攀枝花商业银行自贡分行开业运营，新发展小额贷款和融资担保公司4家，全年新增信贷投放83.2亿元、增长22.4%，增幅为近年最高。行政体制改革不断深化，食品药品监管体制改革全面完成，行政审批事项保持全省最少，193项权力事项下放乡镇。事业单位分类改革有序推进。基层医疗机构运行新机制进一步巩固，县级公立医院取消以药补医改革全面实施。

　　开放合作不断深化。积极推进川南一体化发展，与川南各市发起组建了向家坝灌区建设开发公司。积极支援“4·20”芦山地震抢险救援，对口援建荥经县恢复重建工作有序开展。健全招商激励机制，强化行业招商责任，利用全国机械百强和汽车30强发布会、西博会等平台，加大项目招商力度，全年引进到位市外资金590亿元、增长22.9%，新签约5亿元以上项目52个，投资32亿元的川南通用航空产业基地项目签约落地。强化外资引进，支持企业拓展外贸市场，全年实际到位外资2280万美元、增长13.4%；进出口总额突破10亿美元，其中出口6.16亿美元、增长19%。

　　过去一年，我们大力加强社会建设，切实强保障、惠民生，群众生活持续改善

　　民生工程深入实施。幸福自贡建设扎实推进，各类细胞创建活动广泛开展，14件实事全部办理落实。加强职业技能培训，强化高校毕业生等重点群体就业援助，新增城镇就业3.9万人，城镇登记失业率控制在4.2%以内。城乡养老、医疗等五大保险参保面继续扩大，保障水平稳步提高，近8000名被征地农转非人员社保问题妥善解决，城乡低保月人均补助提高到205元和90元。改扩建养老服务站所19个，农村五保集中供养率达65.3%。住房保障力度加大，首次将农民工纳入城镇住房保障范围，新开工保障性安居工程9771套、竣工9825套，改造棚户区4851套，完成农村危房改造8981户，住房公积金为3万户职工家庭提供购房资金支持12.5亿元。加大开发式扶贫力度，帮助5万多贫困群众实现脱困。解决了19万农村人口的安全饮水问题。建成标准化农贸市场8个。城乡居民收入分别达到21489元和8961元、增长10.5%和12.6%。物价总水平保持稳定。

　　社会事业协调发展。推进义务教育均衡发展，建成义务教育标准化学校40所，育才小学迁建、绿盛南湖分校竣工投用，富顺二中改扩建、荣县新城中学建设加快推进，大安区义务教育均衡发展试点工作通过省评估验收。基础教育课程改革深入推进，教师队伍建设不断加强，教育教学质量稳步提升。学前教育、职业教育、高等教育、特殊教育协调发展，市职业教育集团成功组建。卫生事业加快发展，建成三甲疾控中心1所、二甲中医院3所、标准化乡镇卫生院和社区卫生服务中心95个，四医院住院大楼和消毒中心大楼、富顺县人民医院迁建等项目加快推进，政府办基层医疗机构全部设立中医科，城乡居民电子健康档案建档率达96%。精神文明建设不断加强，成功创建省级文明城市。市文化艺术中心文化馆开工建设，一批示范乡镇综合文化站、公共电子阅览室、社区书屋建成投用，有线数字电视整体转换全面完成；“文明新盐都·快乐自贡人”系列文化活动蓬勃开展，市第十四届艺术节精彩纷呈，一批优秀文艺作品获“群星奖”等国家级大奖；历史文化名城保护加强，新增全国重点文物保护单位5处，自贡世界地质公园荣膺“中国最美地质公园”。全民健身活动深入开展，市第十二届运动会成功举办。低生育水平持续稳定。第三次全国经济普查有序开展。《自贡市志》出版发行。外事、档案、防震减灾、气象、人防、民族、宗教、侨务、对台、邮政、妇女儿童、老龄和残疾人工作取得新成绩。

　　社会管理不断加强。社区治理体系不断完善，建立了城市社区“三分离”自治机制和“三委两站一室”管理服务模式，建成新型农村社区133个。健全社会稳定风险评估制度和“大调解”工作体系，进一步畅通群众诉求渠道，落实信访积案领导包案和接访下访制度，信访总量持续下降，新星源公司实现平稳有序重组。应急管理不断加强，市应急联动及公安信息指挥中心基本建成。加强治安防控，严打违法犯罪行为，命案破案率100%，社会治安秩序良好，市公安监管中心建成投入使用。“六五”普法全面推进，全民法治意识得到增强。扎实开展安全生产大检查，安全生产形势稳定向好。食品药品安全稳定可控。“双拥”共建、国防动员和民兵预备役工作不断加强。

　　各位代表，过去一年，我们能在极为错综复杂的形势下取得上述成绩，实属不易。特别是面对保增长的巨大压力和迈千亿的艰巨任务，市委常委会每季度听取经济运行情况汇报，及时研究经济运行中带趋势性、关系全局性的重大问题，为我们做好经济工作提供了坚强领导；市人大、市政协积极组织开展视察调研，提出意见建议，为我们做好经济工作提供了大力支持；各区县、各部门和有关方面发扬连续奋战精神，迎难而上，全力作为，为全市保增长发挥了决定性作用。在此，我代表市人民政府，向各位代表、政协委员，向全市广大干部群众、政法干警、驻市解放军和武警、消防官兵，向各民主党派、工商联、人民团体，以及所有关心支持我市发展的各界人士，表示衷心的感谢，并致以崇高的敬意！

在总结成绩的同时，我们也清醒地看到，由于宏观经济下行影响，全市经济增速同比放缓，居民收入等部分指标未能实现预期目标，发展中还存在一些突出问题：一是经济增长动力不足，特别是工业增速回落明显，投资总量偏小，项目带动能力不强，保持经济持续较快增长的压力较大。二是经济增长质量和效益不高，特别是产业结构不合理，企业创新能力、盈利能力和竞争力不强，推动转型升级的压力较大。三是财政保障能力不强，特别是收入总量不大、质量不高，加之刚性支出剧增和国家、省转移支付政策调整，保障财政支出的压力较大。四是促进群众持续增收的渠道和办法不多，特别是部分低收入群众的生活仍然比较困难，改善民生的压力较大。这些问题，需要我们在今后工作中努力加以解决。

2014年工作安排

　　2014年是全面深化改革的开局之年，是实施“十二五”规划的关键之年。我们面临的宏观经济形势依然复杂严峻，世界经济仍处在缓慢复苏中，不稳定不确定因素仍然存在。全国经济进入增长速度换档期、结构调整阵痛期和前期刺激政策消化期，增长动力深刻转换，经济运行仍然存在下行压力。我们面临着新的发展要求，就是要按照中央和省委经济工作会议的部署，统筹做好稳增长、调结构、促改革、惠民生各项工作，既要保持合理的经济增速，又要加快转变经济发展方式、调整经济结构，努力提高经济增长的质量和效益。面对新形势、新要求，我们要进一步坚定发展信心，始终保持奋发进取、主动作为的工作状态，抢抓机遇、破难攻坚，凝心聚力、加快发展，努力开创全市经济社会发展新局面。

　　政府工作的总体要求是：全面贯彻落实党的十八大、十八届三中全会和中央经济工作会议精神，用改革统领经济社会发展全局，坚持科学发展、加快发展的工作指导思想，牢牢把握“稳中求进、进中求好”工作基调，深入实施“三大发展战略”，以提高经济增长质量和效益为中心，以转型升级为主攻方向，以项目建设为抓手，着力产业支撑和投资拉动，着力调整优化经济结构，着力加快新型城镇化，着力保障和改善民生，努力实现经济社会快速健康发展，更高水平推进“五个自贡”建设，为加快构建区域中心城市、全面建成小康社会奠定坚实基础。

　　主要指标安排是：地区生产总值增长10%；规模以上工业增加值增长10.5%；固定资产投资增长15%；社会消费品零售总额增长12%；地方公共财政收入增长11%；单位地区生产总值能耗下降完成省下目标；城镇登记失业率控制在4.5%以内；城镇居民人均可支配收入增长10%；农民人均纯收入增长11%。

　　一、做大做强产业，进一步提高发展质量

　　大力实施“产业促进年”，坚持调存量与扩增量并举，主要依托扩增量调结构，推动产业转型升级、提质增效，加快“产业自贡”建设步伐。

　　加快工业转型升级。推动传统产业改造提升，加快机械制造高端化步伐，建成商用车零部件生产基地一期工程，力争开工高精铜深加工项目；推进盐向深加工、盐化工向精细化工延伸，加快鸿化整体迁建；促进食品工业品牌化、集团化发展，实施西南（自贡）无水港油脂加工等项目，打造特色食品品牌。做好建材行业落后产能淘汰工作。大力发展战略性新兴产业，依托重点企业壮大节能环保装备制造业，实施东锅公司气化炉产业化平台、运机节能环保型成套输送装备产业化等项目；依托科技创新大力发展新材料产业，实施中天胜公司1万吨特种聚酰亚胺树脂及复合材料产业化、晨光氟硅新材料产业化等项目；积极培育发展生物医药、信息技术产业，加快建设中新医药产业园、旭阳药业现代中药产业园；战略性新兴产业增加值占比提高0.5个百分点以上。加强园区建设，完善园区基础设施和公共服务平台，着力打造千亿国家高新区，支持区县工业园区建设，规划建设大安工业园区，园区新增承载能力5平方公里；引导园区产业集群发展，强化入园项目联审，建立项目追溯和退出机制，提高投资强度和产出能力、就业能力、纳税能力，全年新入驻项目35个，主营业务收入达到1100亿元以上。强化企业培育，坚持抓大不放小，实施企业梯次培育计划，帮助企业拓展市场，落实各项扶持政策，引导企业建立现代企业制度、实施品牌战略，培育优秀企业家团队，支持发展产业联盟，提升企业核心竞争力，新增主营业务收入30亿元企业1户、10亿元企业2户、规模企业30户。

　　大力发展现代服务业。以省级服务业综合改革试点为契机，加强服务业政策引导，力促服务业发展提速、比重提高、水平提升，服务业增加值增长11%。提档升级商贸物流业，围绕构建区域现代商贸物流中心，加快川南五金城、汽车配件市场、华商国际城、西南（自贡）无水港等重大项目建设，开工建设汽车商贸会展中心。加大建成专业市场培育力度，强化市场业态管理，新培育10亿元以上专业市场4个。加快发展文化旅游业，围绕打造旅游目的地，加快卧龙湖国际旅游度假区、魔幻时光乐园建设，力争盐卤浴主体项目建成投用，办好第20届自贡灯会，发展古镇观光、乡村休闲旅游，旅游综合收入增长15%以上。着力培育新兴服务业，围绕与生产生活新需求相配套，大力发展研发设计、信息服务、金融保险服务业，培育发展电子商务信息平台和结算平台，稳步提升服务业层次。积极扩大消费需求，认真落实促进消费的政策措施，优化消费环境，组织开展各类促销活动，加快培育健康养老、家政服务、文化娱乐等消费热点，扩大住房、汽车等大宗消费，发展批发零售、餐饮住宿等传统消费。

　　积极发展现代农业。加快转变农业发展方式，促进农业提质增效，粮食产量稳定在125万吨以上，农业增加值增长3.5%。加快特色产业基地建设，走种养结合、粮经复合、生态循环、休闲观光发展之路，稳定粮食播种面积，成片建设优质粮食、蔬菜、畜禽、林果、水产特色产业基地，重点打造7个规模上万亩、产值上亿元的现代农业示范园；加强农业基础建设，实施高标准农田、土地整理、小农水等农业项目，提升综合生产能力。完善现代农业经营体系，加快西南粮油及农副产品物流加工产业园和荣县农产品加工园、富顺农产品贸易加工物流园建设，大力培育新型农业经营主体，提升龙头企业、专合组织、社会化服务组织的支撑作用，实施龙头企业技改扩能项目22个，发挥农村经纪人、专业大户、家庭农场的基础作用，发展集体经营、股份合作等经营方式，鼓励工商企业、社会资本投资农业。强化农产品质量安全，健全农业标准化生产体系，支持创建无公害农产品、绿色食品、有机食品和地理标志农产品等品牌，保障“舌尖上的安全”。

　　二、强化项目带动，进一步增强发展后劲

　　坚持依托项目调结构、增后劲、惠民生，推进253个重大项目建设，确保完成投资300亿元，固定资产投资完成600亿元。

　　聚力项目建设。力促在建项目提速，落实项目三级责任制，强化项目推进协调，加快内威荣、自隆高速公路等131个续建重大项目建设，确保海川公司燃机配件制造等50个项目投产投用。力促项目开工，进一步创新审批机制，落实重大项目并联审批和代办领办服务，力促川南通用航空产业基地等84个重大项目开工建设，力争开工内自宜城际铁路。着力解决项目推进中的突出问题，加大土地报征和供给力度，缓解土地供需矛盾；用好国有资本投资项目允许非国有资本参股政策，拓展政府性项目资金筹措渠道；加强银企协调，多渠道帮助项目业主解决融资困难。

　　优化投资结构。切实发挥财政资金的导向作用，支持企业加大产业投入力度，着力推进133个重大产业项目建设，全年工业投资完成175亿元以上。继续加强基础设施投资，着力推进64个基础项目建设，力争完成投资70亿元。突出企业投资主体地位，最大限度缩小审批和核准范围，放宽准入门槛，优化投资环境，激发和释放民间投资活力。

　　全力储备争取。把握国家和省政策投向，做好铁路、棚户区改造、城市基础设施、节能环保等领域项目的包装储备，完善重大项目库。加快乐自泸铁路、自泸大件路、沱江航道升级改造和成自泸城际铁路、富顺-南溪高速公路前期工作，完善项目规划、可研、选址、环评等前期手续，提高项目成熟度。强化对上衔接，力争更多项目纳入省重点项目、得到中央和省预算内资金支持。

　　三、加快新型城镇化，进一步释放发展潜力

　　坚持以人的城镇化为核心，走形态适宜、产城融合、城乡一体、集约高效的新型城镇化道路，不断提高城镇化质量水平，城镇化率提高1.6个百分点以上。

　　有序促进农业转移人口市民化。出台农业转移人口市民化实施办法，全面放开进城落户限制，推行流动人口居住证制度，配套完善进城农民农村权益保障和城镇就业、社会保障等政策，扩大进城农民工住房保障范围，加快探索城乡社会保险转移接续机制，使进城农民与城镇居民享有同等权益。探索建立农民自愿放弃宅基地补偿机制，保障农民宅基地用益物权，让农民愿进城、能进城、留得住。

　　推进城乡一体发展。加强重大基础建设，加快乐自高速自流井连接线、成自泸高速大安连接线建设，力争开工成自泸高速沿滩连接线、内威荣高速荣县连接线、自隆高速富顺连接线。全面完成小井沟水利工程移民对接安置，确保如期下闸蓄水，加快狸狐洞水库建设，力争开工建设楼房湾、大坡上水库；完成仙市220千伏等3个输变电工程，实施4G通信网络建设。推进新区开发，坚持尊重自然、顺应自然、天人合一的理念，高标准推进南部、西部和东北部新城开发，启动东部新城起步区建设，建成卧龙大道一期工程、沙坪南北干道，完成自贡客运总站提升改造，开工建设东部新城公交客运枢纽站。积极推进荣县、富顺县城新区开发，提升城市品质，增强集聚辐射能力。加快小城镇建设，整合资源项目，重点推进4个省级示范镇和一批市级试点镇建设，加强历史文化名镇创建，打造一批规模适度、功能配套、优美舒适、各具特色的小城镇。采取有效措施，确保场镇集中供水安全。连片推进新村建设，以构建产村相融、四级居住新形态为载体，加大力度推进新农村综合体、新村聚居点、新农家大院建设，注重保护村庄原始风貌，配套完善新村基础设施，实施农村电网改造和农村饮水安全工程，新建和改造农村公路260公里，促进农村生活社区化，着力建设美丽乡村、幸福家园。

　　全面实施棚户区改造。遵循“减量、留空、增绿、修复”的原则，注重传承历史文化，分类采取原貌修复、局部更新与整体重构方式，全面推进棚户区改造。加快东兴寺、西山路片区综合改造，完成东兴寺片区居民房屋征收补偿和农房征地拆迁，启动东兴寺大桥立交、铁路桥拓宽等基础设施和安置房建设；完成西山路片区汇园路建设和10万平方米安置房主体建设，开工西山路改造。全面启动光凤片区、汇柴口—火井沱和大安老街等片区改造，完成光凤片区凤凰坝排水主体工程，开工建设光大街片区排水工程，启动汇柴口片区安置房建设和大安老街直管公房居民搬迁。积极推进28个乡镇棚户区改造，力争启动房屋征收补偿。精心做好组织实施工作，确保平稳有序推进。

　　强化生态建设和环境保护。围绕建设“生态自贡”，持续实施“蓝天、绿地、清水、宁静、利废”五大行动。加强城乡绿化，建成卧龙湖湿地公园一期，开工釜溪河复合绿道城区连接段、高峰公园建设。强化节能减排，建成板仓工业污水处理厂一期和84个乡镇生活污水处理工程，加快推进沿滩生活污水处理厂一期及贡井、荣县、富顺生活污水处理厂二期工程建设。引导企业采用先进生产工艺，鼓励发展循环经济。倡导低碳生活理念，做好公共机构节能降耗工作。加强饮用水源地保护，严格实施小井沟、双溪水库生态功能区保护和建设控制规划，持续推进釜溪河流域综合治理。加大城区扬尘治理力度，稳步改善环境空气质量。深化城乡环境综合治理，实施莲花垃圾处理厂封场一期工程、城区垃圾压缩中转站建设，积极推行市政设施维护、环卫作业、园林绿化养护市场化，持续开展“五乱”治理，推进富顺县及一批镇村省级环境优美示范创建工作。

　　四、大力改革创新，进一步激活发展动力

　　把改革创新融入经济社会发展各个领域和环节，积极稳妥推进重点领域改革，强化创新驱动和开放引领，不断增强发展活力。

　　深化重点改革。以激活农村产权为重点深化统筹城乡综合配套改革，进一步做好农村产权确权登记颁证工作，全面实施深化“双挂钩”试点，稳妥推进用地制度改革，探索建立农村集体土地三级所有权管理制度，抓好统筹城乡综合配套改革试验示范区建设，建成市农村产权交易中心，规范开展集体经营性建设用地使用权、林权、承包经营权的流转和交易。以增强市场主体活力为取向深化经济领域改革，大力发展非公有制经济和混合所有制经济，鼓励社会资本通过特许经营等方式参与城镇基础设施建设和运营，支持非公有制企业参股、控股国企改革和政府投资项目；改进预算管理制度，扩大“营改增”试点范围，健全政府债务管理及风险预警机制；完善金融服务体系，支持自贡市商业银行跨区域发展，积极推进城区农村信用社改革工作，新引进域外金融机构1家，鼓励符合条件的民间资本发起设立民营银行；推动聚酯等国有破产企业资产处置和重组，做好市糖果厂等5户企业破产终结，完成煤矿企业兼并重组。以促进职能转变为重点深化行政体制改革，加强政务服务体系规范建设，推进项目审批管理机制创新，进一步清理规范非行政许可审批事项，建立统一规范的公共资源交易平台；加强行政执法管理，规范自由裁量权行使；完成市、县政府部门机构改革工作；有序推进事业单位分类改革。以更好满足群众需求为目标深化社会领域改革，落实教育课程改革和考试招生制度改革措施，探索建立现代学校制度、校长教师交流轮岗制度，积极运用信息化手段扩大优质教育资源覆盖面；完善公益性文化事业单位用人机制和分配机制，加大政府购买文化服务力度；加快县级公立医院综合改革，扩大民办医疗机构纳入医保定点范围，全面实施城乡居民大病保险；鼓励社会力量办学、办医和参与公共文化服务体系建设。

　　加强创新驱动。以科技创新为重点，着力完善技术创新体系，加快建设“创新自贡”。推进南岸科技新区建设，新增承载能力1平方公里，开工建设高新孵化园二期工程。加强创新平台建设，新增省级以上企业创新平台2个，新建企业研发机构10个。强化重点科技攻关，组织实施重大科技项目80项，努力攻克一批产业关键技术。加强创新人才培养和引进，充分激发人才创造活力。加速科技成果转化，完善成果转化金融服务体系，重点实施芳纶Ⅲ先进复合材料产业化等18个转化项目，新培育国家高新技术企业2户，高新技术产业增加值比重达24.5%以上。

　　推进开放合作。完善招商引资工作机制，强化区县、部门招商责任，注重发挥企业招商作用，增强招商引资合力；创新招商引资方式，积极搭建招商载体和平台，加大产业链招商、集群式承接工作力度，做深做细项目前期工作，努力引进一批重大项目，新签约5亿元以上项目25个，到位市外项目资金600亿元；加强跟踪服务，力促签约项目落地建设，提高项目履约率。加强区域合作，主动融入成渝经济区、长江经济带，借势借力发展；积极推进川南一体化，推动组建川南城际铁路公司，提升基础建设、资源开发、产业配套等领域合作实效。加强对外贸易，争取设立综合保税区，培育重点出口企业，拓展节能环保装备、新材料产品外销市场。

　　五、持续改善民生，进一步共享发展成果

　　坚持保基本、兜底线，深入推进“十项民生工程”和“幸福自贡”建设，着力解决群众最关心、受益最直接的民生难题，不断增强群众幸福感。

　　多渠道增加群众收入。健全促进就业和鼓励创业机制，加强职业技能培训，多渠道开发就业岗位，促进高校毕业生、城镇困难人员、退役军人就业，新增城镇就业3万人，鼓励群众通过勤劳致富改善生活。认真落实收入分配改革的政策措施，健全工资决定和正常增长机制，扩大企业工资集体协商制度覆盖面，完善最低工资和工资支付保障制度。鼓励和引导农民以土地承包经营权、宅基地使用权、林权等开展股份合作，多渠道增加农民收入。加强价格监控，稳定物价水平。

　　完善社会保障体系。继续扩大养老、医疗、失业、工伤、生育保险覆盖面，稳步提高保障水平。整合资源加快城乡一体的社会保障制度建设，健全居民养老保险多缴多得激励机制和待遇正常调整机制，完善城乡社会保险制度衔接、转移接续政策。完善社会救助体系，落实社会救助标准与物价上涨联动机制，稳步提高最低生活保障补助水平。加强养老服务体系建设，大力发展社会化养老。健全住房保障体系，加强住房公积金管理，探索公租房共有产权制度，新开工保障性安居工程16845套、竣工7545套。

　　全面发展社会事业。优先发展教育，统筹城乡义务教育均衡发展，加强偏远农村教育工作，完成40所义务教育学校标准化建设；加强教师队伍建设，全面推进素质教育，提高教育教学质量；强化学前教育，新建一批高品质幼儿园；实施职业教育集团化办学，力争开工建设四川康复职业学院新校区，支持四川理工学院创建理工大学。繁荣文化事业，围绕建设“文化自贡”，完善文化繁荣发展措施，健全现代公共文化服务体系，加快市文化艺术中心文化馆建设，推进数字图书馆建设，建成84个社区书屋；继续实施文化惠民工程，促进群众文化活动蓬勃开展；加强文艺创作，打造一批优秀文化作品；加强文化遗产保护、开发和利用；推进彩灯文化产业园建设，鼓励文化企业做大做强。加强卫生事业，完善医疗卫生服务体系，加快推进市一医院儿童住院楼、全科医生临床培养基地、川南职业病防治医院建设，力争开工建设卧龙湖康疗中心，建成区域精神卫生中心，基层医疗机构标准化率达到90%以上；实施区县疾控中心标准化建设，提高公共卫生服务水平。统筹发展其它事业，广泛开展全民健身活动，组团参加省第十二届运动会，积极发展体育产业。认真执行计划生育政策，稳定适度低生育水平。维护妇女儿童合法权益，做好老龄工作，关爱残疾人和特殊群体。支持驻市部队建设，深化“双拥”创建。完成第三次全国经济普查。加快档案数字化建设。加强防震减灾、人防、气象、邮政、地方志、外事、民族、宗教、侨务、对台等工作。

　　不断创新社会治理。充分发挥政府主导作用，强化系统治理、依法治理、综合治理和源头治理，进一步完善社会治理体系，提升社会治理能力。加强基层治理体系建设，完善城市新型社区管理服务平台，新建120个农村社区，充分发挥村（居）民自治作用。完成第九届村（居）民委员会换届选举工作。加强社会组织培育发展，将适合由社会组织提供的公共服务和事务工作交由社会组织承担，激发社会组织活力。加强社会稳定风险评估，完善矛盾纠纷排查调处机制，拓展网上信访受理渠道，落实重要信访市县领导包案制，把涉法涉诉信访纳入法治轨道解决，探索建立涉法涉诉信访依法终结制度，有效预防和化解各类社会矛盾。落实安全生产责任制，健全隐患排查治理体系，强化煤矿、危化品、道路交通、建筑施工等重点领域安全监管，坚决遏制重特大事故。实施最严格的食品药品监管，充实基层监管力量，严厉打击制假售假行为，营造放心消费、安全消费的良好环境。加强应急指挥平台和区域应急联动机制建设，提高应急处置能力。深化社会治安防控“六网”建设，严厉打击各类违法犯罪，切实增强群众安全感。

　　切实加强政府自身建设

　　2013年，我们严格依法行政，自觉接受市人大及其常委会法律监督、工作监督和市政协民主监督，人大议案、代表建议和政协提案全部办复，办理质量稳步提高。加强与各民主党派、工商联、无党派人士的联系，自觉接受新闻舆论监督。坚持科学民主决策，主动公开政府信息。推进行政权力依法规范公开运行平台建设，市、区县行政权力实现网上运行。出台了改进作风、密切联系群众11条规定，深入治理庸懒散奢行为，大力精简文件会议，规范政务行为，干部作风进一步转变。强化行政监察和审计监督，完善了土地及矿权出让、工程招投标、政府采购、国有资产转让等制度，开展公务用车和商业预付卡专项治理，推行“三公”经费公开，严肃查处违纪违法案件，廉政建设进一步加强。同时，我们也清醒地认识到，与形势变化、发展要求和群众期待相比，政府自身建设还存在差距和不足，少数干部思想解放不够，开拓进取精神不强，在解决矛盾问题上创新的办法不多；个别部门和工作人员服务意识不强，办事效率不高，行政效能有待进一步提升。

　　新的一年，我们将紧紧围绕新的使命和任务，不断加强政府自身建设，努力提高推动发展的能力，进一步树立勇于担当、勤政廉洁、务实高效的政府形象。

　　——大力解放思想，在推动改革发展上谋求新作为。始终把解放思想作为政府工作的不竭动力，牢固树立发展意识、改革意识、创新意识，紧紧围绕经济建设中心任务，一心一意谋发展，聚精会神搞建设；紧紧围绕突出矛盾和问题，勇于突破思维定势和观念束缚，善于集中民智和学习借鉴，以开放的眼界、鼎新的锐气谋划工作、破解难题，不断增强改革发展的实际成效。

　　——严格依法行政，在建设法治政府上迈出新步伐。始终把依法行政作为政府工作的基本准则。自觉接受市人大及其常委会法律监督、工作监督和市政协民主监督，广泛听取社会各界的意见建议，依法公开政府信息。完善重大行政决策机制，坚持重大决策出台前向人大报告。坚持用制度管权管事管人，完善重点领域、关键环节权力行使及监督的制度设计。强化依法治理意识，提升依法治理能力，推动形成办事依法、遇事找法、解决问题用法、化解矛盾靠法的法治环境。

　　——践行执政为民，在密切联系群众上展示新形象。始终把执政为民作为政府工作的根本取向，深入扎实开展党的群众路线教育实践活动，坚持问政于民、问需于民、问计于民，了解群众诉求，关心群众疾苦，坚持反对形式主义、官僚主义、享乐主义和奢靡之风，着力解决人民群众反映强烈的突出问题。健全领导干部直接联系和服务群众制度，拓展联系方式和交流沟通平台，推进联系群众制度化、常态化，进一步增进与群众的血肉联系。

　　——弘扬求真务实，在提升行政绩效上实现新突破。始终把求真务实作为做好政府工作的重要保证，大兴调查研究、真抓实干之风，倡行“一线工作法”，加强重大决策、重大项目、重点目标落实的督促检查，着力强化比落实、比实干、比实效的鲜明工作导向。健全工作责任制和绩效考评机制，突出考评结果运用，全面推行标准化服务，深入开展治理庸懒散实施效能问责行动，不断提高行政效能和执行力。

　　——厉行勤俭节约，在建设廉洁政府上提升公信力。始终把勤俭节约作为政府公务行为的基本要求，认真贯彻执行《党政机关厉行节约反对浪费条例》，全面规范财政资金管理、公务接待、公务用车、会议活动等政务行为，全面实施公务卡制度，扩大“三公”经费公开范围，切实降低行政运行成本。进一步落实党风廉政建设责任制，严查违纪违法行为，努力以党风廉政建设的实际成效提升政府的公信力。

　　各位代表，风正千帆疾，心齐万事兴。全面深化改革的东风正吹向盐都大地，全面建成小康社会的宏伟目标激励我们奋勇前进，让我们在市委的坚强领导下，振奋精神、锐意开拓，同心同德、拼搏实干，为推进自贡次级突破加快崛起而努力奋斗！
